# Determinants of demand for condoms to prevent HIV infections among barmaids and guesthouse workers in two districts, Tanzania

**DOI:** 10.1186/s13104-015-1621-y

**Published:** 2015-11-02

**Authors:** Godfrey M. Mubyazi, Amon Exavery, Filemoni Tenu, Julius J. Massaga, Jovitha Rugemalila, Hamisi M. Malebo, Victor Wiketye, Emmanuel A. Makundi, Joyce K. Ikingura, Adiel K. Mushi, Sia E. Malekia, Abubakary Mziray, John W. Ogondiek, Amos Kahwa, Mwanaidi M. Kafuye, Mwelecele N. Malecela

**Affiliations:** National Institute for Medical Research (NIMR), P.O Box 9653, Dar es Salaam, Tanzania; Ifakara Health Institute (IHI), P.O Box 78373, Dar es Salaam, Tanzania; Amani Medical Research Centre (MRC), P.O Box 81, Muheza, Tanzania; National Bureau of Statistics (NBS), P.O Box 796, Dar es Salaam, Tanzania; NIMR Ngongongare Research Station, Usa River, P.O Box 514, Arusha, Tanzania; Muhimbili Medical Research Centre, P.O Box 5004, Dar es Salaam, Tanzania

**Keywords:** HIV/AIDS, Condoms, Poverty, Social-marketing, Stigma, Risk behaviour, Tanzania

## Abstract

**Background:**

Condoms are scientifically recommended as potential products for preventing infections attributable to human immuno-deficiency viruses (HIV). However, evidence on factors leading to their inadequate use in developing countries is still scanty. This paper reports an exploratory study of factors constraining condoms use in Tanzania from the perspectives of barmaids, guest-house workers and retailers.

**Methods:**

Data were collected in two districts—Mpwapwa in Dodoma Region and Mbeya Rural in Mbeya Region—between October and December 2011, using structured interviews with 238 individuals including barmaids, guesthouse workers and 145 retailers. Data analysis was performed using STATA 11 software.

**Results:**

Awareness about condoms was high among all study groups. Male condoms were more popular and available than female ones. A considerable proportion of the barmaids and guesthouses were disappointed with condoms being promoted and distributed to young children and disliked condom use during sexual intercourse. Accessibility of condoms was reported as being lowered by condom prices, shortage of information concerning their availability; short supply of condoms; some people shying away to be watched by children or adult people while purchasing condoms; retailers’ using bad languages to condom customers; occasionally condom shops/kiosks found closed when they are urgently needed; and prevailing social perception of condoms to have low/no protective efficacy. Regression analysis of data from barmaids and guesthouse-workers indicated variations in the degree of condom acceptability and methods used to promote condoms among respondents with different demographic characteristics.

**Conclusion:**

A combination of psychosocial and economic factors was found contributing to lower the demand for and actual use of condoms in study communities. Concerted measures for promoting condom use need to address the demand challenges and making operational research an integral element of monitoring and evaluation of the launched interventions, hence widening the evidence for informed policy decisions.

**Electronic supplementary material:**

The online version of this article (doi:10.1186/s13104-015-1621-y) contains supplementary material, which is available to authorized users.

## Background

### Overview of global situation on HIV/AIDS burden and risk exposures

The ‘human immune-deficiency virus (HIV)’ responsible for causing the ‘acquired immune deficiency syndrome (AIDS)’ is an epidemic problem all over the world despite the some good reports coming out indicating progress in the reduction of the new infections and prevalence in a number of countries. United Nation’s Report reveal that by end of 2012, it was estimated that globally 35.3 (32.2–38.8) million people were living with HIV [1]. Reports consistently indicate the greatest burden of HIV/AIDS being borne by least developed (and mostly poor) countries while sub-Sahara Africa (SSA) has remained the most affected region [2]. Targeting to implement health interventions to areas where the HIV/AIDS epidemic is much more concentrated is greatly recommended, and this includes identification of the groups at most risk of the infections and who are most vulnerable of the epidemic so that they can use appropriate preventive such as condoms and treatment products such as antiretroviral (ARV) drugs, coupled with proper health education and counseling services [1, 3].

Generally, a wide literature indicates studies carried out in different countries having found male and female individuals alike being at risk of contracting HIV infections, but the degree of the risk of exposure to such infections sometimes varying by one’s sex/gender, socio-economic characteristics (including age, level of education, race, income status and decision-making power relations) as well as one’s place of residence [4–6]. Nevertheless, data from some primary research, systematic reviews and meta analyses have somewhat revealed contradictory evidence regarding the risk of exposure or vulnerability to HIV/AIDS among the populations. The majority of older studies and more recent ones have established that women/females have been more vulnerable than men; poor people being more vulnerable as compared to wealthier ones; and young people especially those in the age group of 15–24 years being more vulnerable as compared to population belonging to other age groups [3, 5]. Reports from latest studies with more stratified analyses using data gathered from different SSA countries that focused on the association between HIV/AIDS prevalence and population’s socioeconomic statuses (SES) have also found HIV/AIDS to be: (1) concentrated among the higher SES individuals in the majority of SSA countries, but among the individuals living in poor households only in Swaziland and Senegal; (2) concentrated among wealthier men and women as compared to poorer ones; (3) concentrated among the poor in urban areas, but among the wealthier adults in rural areas of Kenya, Uganda and Zambia; and (4) concentrated among the urban residents than among the poor residents [7]. The vulnerability to HIV infections among specific groups due to factors relating to their occupational exposures also continues being reported, and among the risk exposed groups are the people working in bars, restaurants, guesthouses and those working as long-route (e.g. cross-regional or cross-country) drivers [8].

### Overview of global strategies for prevention of HIV/AIDS

Countries have striven for many years now for fighting the spread of HIV/AIDS through innovative approaches that are aimed at preventing new infections and their transmission to the AIDS free population groups [5]. Promotion of condoms has been viewed and continues being advocated as one of the most effective strategies towards achievement of the latter policy goal so long as this is done along with promotion of behaviour change towards the control of sexually transmittable infections/diseases (STIs/STDs) other than HIV/AIDS [1, 3, 5, 9]. More specifically, the promotion of people to make effective use of condoms is emphasized as a priority if well coupled with such measures as sensitizing all sexually active individuals to reduce the number of sexual partners, practice abstinence (especially among the young people); males undergoing male circumcision; if decision to use condoms is made, then such products should be used correctly and consistently [1, 2, 10, 11]. Other measures recommended include the sexual partners striving to know and disclose their HIV statuses [12]; avoiding excessive use of alcohol; removing fear that condoms are not protective or are harmful if used in cervical parts [8]; perception that condoms remove/reduces sexual pleasure and are non-romantic [5, 13, 14]; and that condoms contain health harmful worms [15]. The benefits of condom use continues being widely acknowledged in the literature. Experts have been suggesting that the consistent and proper use of condoms has potential to help the majority of adolescents, youths and adults in countries of south-eastern Asia and SSA where there is predominance of unprotected hetero-sexual practices [3, 9]. However, scientific debates are still raising the question about the ability of the countries to attain the goal if condoms continue facing stigma leading to their incorrect and inconsistent use among the populations targeted [5, 15, 16].

There is voluminous literature regarding the factors seeming to hamper the utilization of condoms in developing countries and the issue of negative social beliefs about the disease and condom use, therefore, leading to negative attitudes among the potential users who end up avoiding to use condoms during heterosexual moments is recurrently reported [17, 18]. Among the measures considered to be necessary for supporting current strategies/programs aimed at enhancing condom use is research undertakings aimed at establishing evidence on the factors behind the observed low and inconsistent use of condoms. It is emphasized that research should be an integral element of monitoring and evaluation (M&E) of the programs in place in terms of how far such programs are acceptable, accessible, affordable and usable [19].

We have continued witnessing and acknowledging the role of the media working in cooperation with such NGOs as PSI-Population Survey International and government Ministries of Health in the campaigns for enhancing public awareness on condoms and HIV/AIDS prevention in SSA countries and elsewhere around the world. The campaigns have included the actual and wider distribution of condoms and their delivery at free price or at highly subsidized prices and sensitizing community members to use them when they wish to perform sexual actions with their temporary or even casual partners so long as they suspect the possibility of HIV infections [20, 21].

Unfortunately, we continue receiving controversial evidence about the promotion of condom use through wider distributions involving high subsidization or free delivery. Critics argue that this has contributes influence the adolescents to engage themselves into sexual behaviours some of which are risky when a number of them end up not using the commodity as recommended [22]. Other experts in the field of health marketing argue that the promotion of condoms through public education, individual person counseling and advertising really encourages condom use, but the desired impact may not be fully realized if the promotion carried out has not been coupled with condom distribution to make such commodities more readily available and affordable to the intended individuals. That is to say thatthe evaluation reports are still needed to establish evidence on different countries’ experience with the effectiveness of their condom promotion and distribution programs [23, 24].

### HIV/AIDS situation and knowledge gaps regarding condom use in Tanzania

In Tanzania, where the first case of HIV/AIDS was reported in 1983, HIV/AIDS is one of the first two public health problems causing high morbidities and deaths among the population [25]. Records who that by end of 2012, over 1.5 million people in this country were living with HIV/AIDS, with approximately 86,000 new infections, although the national prevalence rate has declined from 7.0 % in 2003/04 to 5.3 % in 2011/12 [26]. Although this rate seems to be relatively lower than that of the hardest hit countries in SSA [27], the latest records indicate the prevalence rate of HIV/AIDS to be 6 % in the country among the people aged 15–49 years [28]. Previous studies have established that barmaids and guesthouse keepers are among the people most at risk of HIV infections and their risk exposure may be the same as that reported for the commercial sex workers [29]. Inadequate and inconsistent use of condoms among the people who are at risk including those having multiple sexual partners and extramarital sex is reported to be a public health challenge [30]. While the prevention of new infections, especially among the adolescents and youths is the first national priority among other HIV/AIDS control strategies [26], there still need for more reports on the acceptability and utilization of condoms by different population groups targeted by the existing HIV/AIDS control programs. The messages coming from systematic studies are likely to help answer the outstanding question and guide the national AIDS control Program (NACP) to plan and implement more effective interventions. Among the evidence needed is that helping to elucidate the determinants of condom supply and demand for condoms and then give feedback to the authorities responsible for planning and implementing desirable interventions [30, 31]. That is why the Global Fund Round 8 Secretariat in Tanzania approved and funded the proposal for the currently reported study attempting to explore, among other things, the demand related determinants of condom use among the barmaids and guesthouse workers in two districts located in two different regions in Tanzania in 2011. Additional findings relating to the school children’s perceptions and utilization of condoms coupled with assessment of their views regarding the availability, affordability and acceptability of condoms in the same study districts have already been documented [31].

## Methods

### Study design and areas

The present study was exploratory in nature and took a cross-sectional survey design, having been undertaken in two districts, namely Mpwapwa that is located in Dodoma Region around central Tanzania and Mbeya Rural (R) located in Iringa Region in the south-western highlands of Tanzania [3, 13]. The term ‘inappropriate use of condoms*’* has been used in this paper refers to irregular and inconsistent use of condoms contrary to public health recommendations [28, 32]. We were motivated to identify the two study districts by the evidence that in both developed and developing countries, some people avoid using condoms due to such causes/factors as personal, social/cultural, geographical or systemic factors. These factors are important as they act as determinants of demand apart from the status of availability of such products that tends to vary from one geographical area to another [32–34]. Thus, given their locations in different regions that are far apart from each other, the reports on these districts showed different socio-economic characteristics and HIV prevalence rates between them [31]. The HIV/AIDS prevalence rates were lately reported to be 9.2 and 3.3 % for Mbeya Region and Dodoma Region, respectively [25]. Nevertheless, specific data for each district was not yet documented officially at the time of the present study was being conceived and designed [31].

### Sampling methods

The study population groups targeted were individual barmaids and guesthouse workers/attendants as regards information relating to their experience with and attitudes towards condom use. But additional data were gathered from the condoms retailers who have been interacting with individual customers of different age groups among whom might be barmaids and guesthouse attendants. Inclusion or targeting of barmaids and guesthouse workers was based on the evidence that these groups are among those most at a higher risk of contracting HIV/AIDS infections, other groups being commercial sex workers and adolescents. The reports reviewed indicate that the risk exposure and vulnerability of these people are contributed by the persuasions they receive for entering into sex actions from several and different bar clients they serve, particularly when they get tempted to receive monetary compensation from unfaithful HIV positive men to engage with them in unprotected sexual intercourses [35, 36]. According to Lwihula and colleagues, barmaids are reported in various research reports as people working in both licensed and unlicensed bars, employed to sell beer at low wages and in the attempt to supplement their meagre income, they involve themselves in sexual relationships with the customers they may not know of their HIV status, for example, truck drivers who may stop overnight, and rich but promiscuous men of the area. Guesthouse workers are faced with the same situation [8].

The condom retailers were targeted as potential condom traders directly interacting with condom customers in the visited study streets, and indeed, a number of them currently do this business in Tanzania [29]. A mixture of random and non-random sampling techniques was adopted to select the study subjects. This depended on the characteristics and number of the respondents targeted to be covered. A multistage sampling system was followed whereby the first step was to identify the districts randomly from the two regions purposefully selected for the study. The districts were selected out of the list of all districts forming each of the respective regions. We wrote names of each region’s district on different pieces of paper and put such papers in the container that was shook strongly and then allowed one piece to be picked out with the name of the district identified randomly for study. A similar approach was used to write the names of the villages for each district and from them a random identification of four pieces with names of the villages for study per district was done. However, the selected villages were identified from the individual wards that were also selected randomly as described for the selection of the districts and villages, and we did so by adopting a same approach as used in past studies [37]. The sample size for bars, guesthouses and condom retail outlets was not determined or estimated in advance because of lack of baseline data/records from the respective study districts and regional authorities. That is, after the research team had failed to secure a list of officially established bars and retailers from the study district authorities, the issue of sample sizes of these populations was left to be known at the end of the study. Until the end of the data collection process, 123 and 115 individuals were enrolled in Mbeya (R) and Mpwapwa, respectively, and these include barmaids and guest house staff groups. Meanwhile, 69 and 36 of the condoms retailers were finally covered in those districts, respectively. The criterion that was relied on for enrolling the participants was that the person concerned had to accept participation wilfully or voluntarily and had to be 18 years or older (Additional files 1, 2).

### Data collection processes

The data were collected using a standard structured interviewer-administered questionnaires and these include the members of the research team and the trained enumerators who had experience with working on public health research projects involving community household surveys. Two questionnaires were used, one used for gathering data from Barmaids and Guesthouse workers and another one used for data gathering from Condoms Retailers. Some of the questions had binary answer options; others allowed multiple responses; a few questions were open-ended. Respondents from the barmaids and guesthouse workers’ side were asked to give their opinions regarding the prices, distribution, and promotion of condoms in their community settings, demand for- and types of- condoms stocked at retail outlets, customer preferences to different types of condoms, and their socio-economic and demographic characteristics. Also, they were asked about retailers’ condom selling behaviour focusing on language used to customers, handling of condoms with other consumer products in their market places, pricing systems, types of condoms preferred to be stocked, and influence of such behaviours on customers’ attitude towards condom use. Furthermore, the barmaids and guesthouse workers were asked about their personal experiences with condom use (i.e. if they personally ever used condoms) and perceptions about condom promotion and distribution to children of school age and general public through the media (Additional file 1). The retailers were mainly asked about the types of the condoms they were selling and affordability of such products; who were the main customers coming to buy condoms from their outlets (shops/kiosks); reliability of the supply of their condoms; customer preferences to different types of condoms; and customers’ attitudes towards condoms and transparency in their condom buying process (Additional file 2).

### Data variables and statistical analysis

The data collected were double-entered immediately after preliminary inspection to correct the necessary errors. Analysis was performed using STATA 11 software. The results computed were executed and displayed as it seemed appropriate i.e. one-way frequency distribution tables, Chi square (χ^2^) tests, multivariate logistic regression analysis (LRA) with interest in adjusted odds ratios (AOR) and P-values at 95 % confidence interval (CI). Statistically significant differences in the observed parameters or frequencies based on the tests conducted were taken to be at a threshold of P ≤ 0.05. The definitions of the outcome (dependent) variables that were included in the LRA depended on type of the questions of interest for the analysis. The respondent’s experience with (1) condom use prior to the present study; (2) engagement in the sexual intercourses with either permanent or temporary partners before; and (3) their perceptions of whether or not condoms could offer protection against HIV infections, formed a list of the outcome variables. Meanwhile, the predictor variables that were fitted in the LRA models include such individual respondent’s characteristics as: age, place of residence, sex, and knowledge about condoms. However, some of the outcome variables used in a particular model were also taken as potential predictor variables in the other model: for instance, perception about the protective efficacy of condoms for HIV infection prevention was a predictor variable used in the model assessing the determinants of one’s previous use of condoms and this was fitted in the same model along with other predictor variables. A multivariate LRA approach adjusted for possible confounders, as similarly employed in the previous analyses discussed elsewhere [28, 31, 38, 39].

### Ethical considerations

The national ethical clearance for the study was granted by the Medical Research Coordinating Committee (MRCC). Regional and district authorities were contacted in advance from the time the study protocol was being developed for the planned study visits, and were informed after the study was approved by MRCC for their notification and reconfirmation of their acceptance of the study to be carried out in their places. The community members were approached at first by contacting their local government leaders who after granting the team permission to a go ahead, they were directly approached and given more explanation on the study’s objectives, implementation strategy, expected benefits, non-paid time for anyone volunteering to participate, and other important ethical issues related to their voluntariness or freedom to participate or decline participate in the study without facing any pressure or cohesion or such negative consequences as penalty for their decision. They were also informed of the plan to disseminate the study findings for policy use and public domain; confidentiality of the information they would give and anonymity of their names, as well as the required condition for signing an informed consent form for those ready to participate. The illiterate ones were allowed to appoint their representatives to sign the form on their behalf or give their thumb print.

## Results

The number of responses or respondents varied, depending on the nature of the questions posed. Therefore, the frequencies/percentages shown in the tables indicate either the number of the responses obtained per specific questions or number of individual respondents to such questions.

### Demographic characteristics of the respondents

Among 238 barmaids and guesthouse workers from both districts, 51.7 % (n = 123) were found in Mbeya (R), 48.3 % (n = 115) were found in Mpwapwa. The majority of the respondents were below 35 years of age; the mean age being 29.4 years (SD = 8.1). Although all the respondents were literate, only 10 % received secondary school education. Only 38 % (n = 89) of the respondents were married. As for the condom retailers, 145 individuals from both districts were covered, and among these 51.1 % (n = 74) were females. Moreover, out of the 145 retailers involved, 52 % came from Mbeya (R) district; the rest came from Mpwapwa district. The education status of the latter respondents was as follows: 53.9 and 44.7 % had completed primary education and secondary education, respectively. The rest had never gone to school (Table 1).

**Table 1:** Demographic backgrounds of barmaids and guesthouse workers interviewed in Mbeya (R) and Mpwapwa Districts, Tanzania in 2011

Demographic characteristics	Barmaids and guesthouse keepers		Condom retailers	
	No.	%	No.	%
Sex				
Male	78	32.9	71	48.9
Female	159	67.1	74	51.1
Total	237	100	145	100
Age group in years				
<35 years	174	73.1	64	48.1
≥35 years	64	26.9	69	51.9
Mean = 29.4, SD = 8.1				
Total	238	100	133	100
District of residence				
Mbeya Rural	123	51.7	69	65.7
Mpwapwa	115	48.3	36	34.3
Total	238	100	105	100
Education				
Never gone to school	28	11.8	2	1.4
Primary school	185	78.1	77	53.9
Secondary school	24	10.1	64	44.7
Total	237	100	143	100
Marital status				
Married (in union)	89	37.7	–	–
Single	98	41.5	–	–
Never got married	49	20.8	–	–
Total	236	100	–	–

### Personal engagement in sexual relations, intercourses and condoms use behaviour

#### Participation in sexual intercourses among barmaids and guesthouse workers

The question about personal experience with engagement in sexual intercourse in the period prior to this study was answered by 236 respondents among whom 98.3 % (n = 233) affirmed while the rest denied. The same question was extended to get specifications on whether or not the respondents personally have ever had (1) permanent sexual partners; and (2) temporary sexual partners. The first question on possessing permanent sexual partners was answered by 236 respondents, among whom 64.8 % (n = 153) affirmed; 32.6 % (n = 77) denied while 2.5 % (n = 6) decided to keep the answer as their own secret. The question about possessing temporary sexual partners was answered by 237 respondents, among whom, 69.9 % (n = 165) affirmed, 29.1 % (n = 69) denied, while 1.1 % (n = 3) keep it confidential to themselves. Another question was about the frequency at which sexual intercourse was shared with temporary sexual partners. Clear answers for this question were obtained from 235 respondents and among whom 54.9 % (n = 129) affirmed to have done so regularly; 34.0 % (n = 80) said to have had done so only occasionally (irregularly); while 11.1 % (n = 26) shied away to disclose their behaviour and kept it confidential to themselves.

The above questions were followed by another question asking the respondents about whether or not they personally have been using condoms while performing a sexual action with their partners. Focus was made on sexing with permanent or temporary partners. With reference to condom usage during sex with temporary partners, the answers were obtained from 237 interviewees, indicating that even the two respondents who did not affirm in the foregoing question about having had shared sex with their partners did actually have ever done so. Thus, among the 237 respondents, 82.3 % (n = 195) testified/affirmed to have been using condoms with such partners; 16.9 % (n = 40) denied; while the rest kept this information confidential to themselves. Table 2 presents the results from LRA in the barmaids and guesthouse attendants’ data case. As shown, those found with the age of 35 years or above (≥35 years) were 89 % less likely to report to have personally entered into sexual intercourse with their temporary partners than the young ones (i.e. those aged <35 years). The age of 35 and above qualifies many to have been married already and therefore sticking to their marital relationships. On the other hand, the married ones were 70 % more likely to report having had sex with the temporary partners than the single ones (AOR = 0.30; 95 % CI 0.01–7.24), contrary to expectations, although as discussed later the older people might be more confident to speak on sexual issues if they trust the investigator; those ever married before but were no longer in marriage bondage were 83 % less likely to report having been in sexual intercourse with such partners than their counterparts who were still in marriage contracts (AOR = 0.17; 95 % CI 0.01–8.23). As for the place of residence, the respondents from the Mpwapwa district were 44 % less likely to report having engaged themselves into sexual intercourse with their temporary partners than their counterparts from Mbeya (R) district (AOR = 0.66; 95 % CI 0.05–8.23). However, no any statistical significant differences were observed in these results.

**Table 2:** Multivariate logistic regression of factors associated with ever having had sex among barmaids and guesthouse workers in Mpwapwa and Mbeya (R) districts, Tanzania (n = 234)

Covariate	Adjusted odds ratio (AOR)	95 % CI	*P*-value
Sex			
Male	1.00	‒	‒
Female	2.71	0.32‒23.20	0.363
Age category (years)			
<35	1.00	‒	‒
≥35	0.11	0.01‒1.52	0.100
Marital status			
Married	1.00	‒	‒
Single	0.30	0.01‒7.24	0.458
Ever married (currently divorced or widowed)	0.17	0.01‒2.57	0.200
District			
Mbeya Rural	1.00	‒	‒
Mpwapwa	0.66	0.05‒8.23	0.750

### Knowledge and perceptions about promotion of condoms to the public and children

The question on types of condoms known was answered by 235 respondents among the barmaids and guesthouse workers. Some indicated to be aware of male or female condoms only and a few others reported to know both types. Knowledge on (1) male condoms only was expressed by 51.9 % (n = 122); (2) female condoms only was expressed by 5.1 % (n = 12) while (3) both types of condoms by 41.7 % (n = 98). The rest 1.3 % (n = 3) were unaware if any of these condoms really existed in their local community settings.

Reports on the sex of the customers who mostly sought to buy the condoms from the shops/kiosks were obtained from 141 retailers out of 145 interviewed. Of those reporting to have confronted the said type of customers, 91.5 % (n = 129) and 4.2 % (n = 6) identified the groups of male and female customers, respectively while the rest, that is, 4.2 % (n = 6) could not recall for them to identify which specific group.

Among 237 barmaids and guesthouse workers who expressed their perceptions about condom promotion by distributing them to adult people and children, only 11.8 % (n = 28) viewed condoms as not being important while the rest were in support. Furthermore, advertising condoms to promote them through mass media and other mechanisms was perceived as being improper if targeting children of school age. From 236 respondents to the question addressing this issue, 31 % (n = 74) felt that promoting condoms to children was inappropriate or socially immoral. Only 2.1 % (n = 5) did not show their stance in terms of whether they were in support or against the move while 66.7 % (n = 157) were in strong favour of the move. Meanwhile, the issue of delivering health education to the public whereby parents/adults and children are listening and/or seeing at the same time through such means as media was perceived differently. Out of 237 respondents, 88 % were in favour of the approach while the rest were against. Those against the move felt that open promotion of condom use among children could risk the children even more by making them enter in premature sex (data not shown).

The question on whether or not condoms have any potential benefit including offering protection against HIV infections was answered by 237 respondents. Among these, 61.2 % (n = 146) were in strong agreement; 24.9 % (n = 59) were weakly agreeing, 6.3 % (n = 15) completely disagreeing, while 7.2 % (n = 17) were indifferent. Chi square test indicated that those reporting to have used condoms previously and believing condoms to offer protection against HIV infections proportionately outweighed those who did not believe so, the observed difference being highly statistically significant (P < 0.001).

The LRA performed using data from both districts combined indicates that female respondents were 52 % less likely to acknowledge having used condoms with their partners before than the male respondents (AOR = 0.48; 95 % CI 0.20–1.15). Those below 35 years of age were 55 % less likely to report having used condoms previously than the elder ones and in this case the difference was found to be statistically significant (AOR = 0.45; 95 % CI 0.20–1.02). The respondents who were still single at the time of the present study were three times more likely to report having used condoms than the ones still in marriage (AOR = 3.73; 95 % CI 1.46–9.51); the observed difference in the latter case was found being statistically significant (P = 0.006). The ever married (but no longer in marriage) ones were also three times more likely to confirm having had used condoms before than those currently in marriage (AOR = 4.86; 95 % CI 1.50–15.78); and as sown the observed difference in the latter case was statistically significant (P = 0.008). As for place of residence, those found in Mpwapwa were 67 % more likely to report having used condoms than their counterparts in Mbeya (R) (AOR = 1.76; 95 % CI 0.81–3.82). In terms of education, those who had at least primary school education (standard 7 leavers) were 33 % more likely to testify having used condoms than those who never went to school at all (AOR = 1.33; 95 % CI 0.48–3.72). Those who accessed secondary education were three times more likely to report having used condoms than those who never went to school (AOR = 3.34; 95 % CI 0.55–20.20). In addition, the respondents who confirmed to possess temporary sexual partners were 47 % less likely to acknowledge to have been using condoms than those who reported to possess permanent sexual partners. Those who did not disclose whether they possessed sexual partners were 91 % less likely to testify having been using condoms than those who reported to possess permanent sexual partners (Table 3).

**Table 3:** Multivariate logistic regression of factors associated with condom use among barmaids and guesthouse attendants during sex in Mpwapa and Mbeya Rural districts, Tanzania (n = 231)

	Adjusted odds ratio (AOR)	95 % CI	P-value
Sex			
Male	1.00		‒
Female	0.48	0.20‒1.15	0.100
Age category (years)			
<35	1.00		‒
≥35	0.45	0.20‒1.02	0.057
Marital status			
Married	1.00	‒	–
Single	3.73	1.46‒9.51	0.006
Ever married	4.86	1.50‒15.78	0.008
District			
Mbeya Rural	1.00	‒	‒
Mpwapwa	1.76	0.81‒3.82	0.150
Education			
Never been to school	1.00	‒	
Primary	1.33	0.48‒3.72	0.586
Secondary	3.34	0.55‒20.20	0.189
Sexual partner type			
Permanent	1.00	‒	‒
Temporary	0.53	0.22‒1.23	0.138
Undisclosed	0.09	0.01‒2.08	0.134

Goodness-of-fit test: Hosmer–Lemeshow Chi square (8) = 11.94 (P = 0.154)

### Types of condoms promoted commercially and target customers’ preferences

Condoms retailers identified at least five brands of condoms that were being sold through their shop/kiosk outlets. They reported the commonly stocked brands to include *Dume* [meaning male], *Lady Pepeta*, *Family*, and *Raha*, and *Rough Rider*. Of all these, the Dume was the reported as most popular, as indicated by 131 (95.6 %) out of 137 respondents. As to whether the condoms sold by the retailers were meeting the target and potential customers’ preferences in the communities visited, answers were obtained from the barmaids and guesthouse attendants who acknowledged to have been using condoms. Among the respondents, 68.7 % (n = 132) believed so; 12.5 % (n = 24) did not believe so while 18.8 % (n = 36) were indifferent. Among those who denied to have ever used condoms before, 51.3 % (n = 20), 10.3 % (n = 4), and 38.5 % (n = 15) gave the same pattern of the responses as those in the foregoing category of answers, respectively. Statistically, the observed difference between those agreeing/believing and those disagreeing/not believing with the fact that the condoms sold by retailers met the preferences of the intended users, tested using Chi square method, was significant [χ^2^(1) = 7.3; P = 0.025]. Among 144 retailers who answered the question about the time seeming to be preferred by majority of the customers who show up to buy condoms, 40.9 % (n = 59) mentioned the evening times (not later than 6.30 pm); 33.3 % (n = 48)—night times (during darkness especially from 7 pm); 19.4 % (n = 28)—no specific time; while the rest include those who mentioned such moments as early morning, afternoon, and around midday, and each of these accounted to <5 % of the interviewees.

### Availability and affordability of condoms sold by retailers at community level

The supply of condoms in general in the study communities was reported as occasionally running out of stock, as indicated by 72 % of the answers from barmaids and guesthouse workers (n = 236) and confirmed by 42 % of the retailers (n = 142). As to whether or not the condoms could be accessed for free in the places visited, only 52 % of all the responding barmaids and guesthouse workers (n = 236) acknowledged to have known such places. In terms of prices of the condom sold by the retailers, 74 % out of 234 respondents among the barmaids and guesthouse workers group perceived the prices as being reasonably affordable; 19 % perceived the prices as being too high and unaffordable to poorest people; while 7 % had no clear answer. Reports about perceived un-affordability of condoms prices among a few customers were confirmed by retailers. Among the 142 retailers who gave responses to the question, 45.8 % (n = 65) testified/affirmed to have regularly been meeting customers asking to be offered condoms for free; 75.4 % affirmed to have met such customers rarely; 18.5 % met such customers very often; while the rest did not recall who frequently they have been meeting such customers. Asked to specify which age group seemed to ask for help with free condoms most, 63.6 % of the responding retailers (n = 142) pinpoint adolescents and teens aged groups (15–24 years); 16.1 % mentioned junior adults aged between 25 and 39 years; 6.9 % identified younger children aged 10–14 years; while the rest 4.9 % could not recall what they were experiencing for them to specify the group, although they admitted to have met them.

### Condoms selling behaviour of retailers and possible implications on condom use

Experience was shared about the undesirable retailers’ condoms selling behaviours that disappoint potential buyers and users of condoms. Among the barmaids and guesthouse group, reports were presented regarding the poor language used by retailers to their customers; time at which the retailers open their outlets sometimes inconveniencing the customers who find the outlets being closed; the way (approach)used by some retailers to handle the condoms as some could be found storing such products poorly by either exposing them to intense sunrays or mixing them with food products or exposing condoms too much (i.e. not covering them) while serving them to the customers who were eventually discouraged to be seen by other people who were around. Only about 10 % of the respondents were dissatisfied with the language used by the retailers and the manner in which condoms were being stored.

### Social stigma about condoms and implications on their actual utilization

Responding to the question requiring them to express themselves how they would feel in case they were to be seen by either a parent/guardian or other adult people while buying condoms, 58 % among the barmaids and guesthouse attendants (n = 144) expressed not doubt; the rest claimed that they would either shy away and therefore refrain buying the condoms or not throwing such commodities away. Similarly, 68 % (n = 144) among the same group of respondents said that they could not worry anyhow, but only if they were seen by adult persons other than their parents/guardians; the rest were open to admit their possible shying away to buy condoms or throwing them in the bush/pit if seen by the said people.

Prevalence of the tendency by some customers shying away to buy condoms openly were confirmed by the retailers. It was revealed by at least 86 % (n = 114) of the retailers that the majority of the customers seem shy to tell the seller openly to give them condoms in the presence of other adult people. Therefore, some of the customers tend to use children for buying condoms on their behalf or use parables while they are at the shop in attempt to inform the seller after noting other people’s presence. Alternatively, buyers decide to write their need for condom on small pieces of paper and pass them to the seller to read secretly and serve them accordingly. Sometimes, the customers ask the seller to pack the condoms secretly to enable them handle such materials as something else not easily noticeable by other people. When asked further about the age group of the customers seeming to be more comfortable/confident when buying condoms in the presence of other people at their selling outlets (shops/kiosks), 77.5 % of the respondents (n = 110) identified adolescents aged 10–24 years; 17.6 % (n = 25) identified people aged ≥25 years; the rest reported to never care to make such follow up for comparison purpose.

As for the perceived protective efficacy of condoms against HIV infections, LRA indicated that among the barmaids and guesthouse workers, those aged 35 years or above were 2.7 times more likely to express their trust in the protective potential of condoms against HIV infections than the younger ones (AOR = 2.74; 95 % CI 0.55–13.58). Both female and male respondents were in same degree of perceiving that condoms offer protective potential (AOR = 1.03; 95 % CI 0.31–3.42). Single ones were 43 % more likely to believe in condoms than those currently in marriage (AOR = 1.43; 95 % CI 0.40–5.07); the ever married before were 2 times more likely to trust in condoms than those currently in marriage (AOR = 2.07; 95 % CI 0.37–11.6); those from Mpwapwa were 68 % less likely to believe so than their counterparts from Mbeya (R) (AOR = 0.32; 95 % CI 0.09–1.07). Moreover, those who had primary school education and those with secondary school education were less likely to trust in condoms than those who never attended any of these levels of education (Table 4).

**Table 4:** Multivariate logistic regression of factors associated with perceived protective potential of condom against HIV infection among bar maids in Mpwapa and Mbeya (R) districts, Tanzania (n = 217)

Covariate	Adjusted odds ratio (AOR)	95 % CI	P-value
Sex			
Male	1.00	‒	‒
Female	1.03	0.31‒3.42	0.958
Age category (years)			
<35	1.00	‒	‒
≥35	2.74	0.55‒13.58	0.217
Marital status			
Married	1.00	‒	‒
Single	1.43	0.40‒5.07	0.579
Ever married (currently divorced or widowed)	2.07	0.37‒11.6	0.406
District			
Mbeya Rural	1.00	‒	‒
Mpwapwa	0.32	0.09‒1.07	0.065
Education			
Never been to school	1.00	‒	‒
Primary	0.64	0.08‒5.35	0.680
Secondary	0.47	0.04‒6.07	0.560

## Discussion

### Supply-related influences on demand for condoms in the study community settings

To start with, we would say that the present study confirms that extramarital affairs are common in Tanzanian communities and therefore any strategy encouraging people to use condoms especially when they share sex with their temporary sexual partners are justified. The present study confirms that in actual sense, condoms of different types and brands are distributed and promoted in Tanzania, but gaps (or imbalances) do prevail between the supply of and demand for such products. The general impression of the results presented above confirms the fact that people may not desire anything they are not familiar with or aware of its benefits and availability. Having information on condom benefits is one thing, but people needing to know where and when such products can be accessed is another thing of great importance in the health care market context. Under the reported situation whereby male condoms are found being the most popular and commonly distributed, the chance for promotion strategy in the system to favour men than women becomes obvious. This means, women may be willing or eager to use condoms as a way of adhering to safe sex recommendation, but may be limited or barred by shortage or lack of female condoms for them take lead in influencing condom use-related decisions at the time of doing sex with men if the latter disagree/decline using the male condoms available in the market and therefore expose the women to risk of HIV infection. The chance of condom use eventually depends on the willingness of men who should be the catalysts for change in risky sexual behaviours. This view is supported by the testimonies from the barmaids and guesthouse workers revealing that already the majority were regularly sharing viginal sexual relationships with their temporary male lovers and among them a considerable proportion was not using condoms. This finding and arguments are supported by reports from earlier studies in East Africa and elsewhere in the least developing world whereby barmaids were found being at a higher risk of contracting HIV through unsafe sex [8, 35]. Also, it can be seen that targeting promotion of condom use among the teenagers and adolescents is imperative since the reports presented from the present study reveal that the majority of the barmaids and guesthouse attendants who reported to have been doing unsafe sex (without using condoms) with temporary lovers were females and younger ones.

Meanwhile, the reported unawareness about condoms availability in the local places as heard from the respondents in this study reflects how the presence of a product in the market does not warrant or guarantee the popularity of such products unless there are more innovative and sustainable efforts for marketing the products through promotional programmes. That is, the findings brings us to see an urgent need for the current and future programmes to sustain or strengthen efforts aimed at marketing condoms more and more. This is possible if the strategies suggested are coupled with (or include) the actual supply/distribution of both male and female condoms -otherwise the story will remain the same—since there is a common adage that ‘*out of sight, out of mind*’. For instance, a multivariate LRA analysis from a study conducted recently in Tanzania found that among the 10–19 year olds adolescents, those who were aware about availability of condoms or have heard about condoms in the places where such products could be reached were more likely to use condoms than those who were not aware [28].

### Perceptions about condoms promotion, prices and use among targeted populations

The degree of condoms use during sexual actions may depend on, among other things, how frequently people engage themselves in sex and with whom, their attitude towards condoms, availability, promotion and pricing of condoms, and consistent and correct use, and quality, of the condoms. Interestingly, in the present study, some of the barmaids and guesthouse workers honestly reported being aware of condoms, having temporary partners with whom they had been sharing sex using condoms, but honestly to have not always/regularly used condoms. The reasons for the non-use of condoms as we have seen include attributes relating to the inadequate supply of condoms in the existing market although the respondents concerned were ready and willing to use such products. Meanwhile, those who were hesitant to openly talk about their sexual relationships with temporary or permanent partners including the experience with condoms use revealed how the stigma against open discussion on sexual issues and condom use has prevailed in the society.

Furthermore, the low awareness on the availability of different types of condoms among the study population shown by the present study respondents mirrors the possibility that the different strategies used by condoms marketers in different district/community settings to promote condoms had different strengths: lack of information on product availability (including condoms) contributes limiting the potential users of condoms to use such products [38]. The differences noted between female and male respondents of different sex in relation to their condoms utilisation behaviour are consistent with the findings reported from other countries [40]. The results from a LRA model using data gathered from barmaids and guesthouse workers indicating the male respondents being more likely to affirm openly and honestly their previous engagement in heterosexual behaviours and using or not using condoms than their female counterparts. This reflects the possibility that in general and in real world situation the women tend to shy away more than men to testify their participation in such practices in Africa and this shyness is likely to have social-cultural root causes [41].

The concern on condom prices limiting the poor people to use condoms as raised by a few respondents is another demand determinant calling for more attention from the authorities or agencies targeting to enhance demand for condoms in the fight against HIV and other STIs as well as in the promotion of child-spacing (including family planning) campaigns. Apparently, the people who request for free condoms from the retailers as noted from the present study represent those who are likely to end up without using condoms during sex if their request for free condoms were rejected purposely for justifiable reasons. Additionally, the expressed dissatisfaction with the approaches used in publicising/advertising condoms to the public irrespective of age-groups is another programmatic and systemic challenge facing the HIV/AIDS control programmes in Tanzania. Therefore, it is a matter of the authorities concerned to re-think on how better/best the conservatives’ mindset could be changed so as to encourage them think positively toward condoms and current/likely condom promotion strategies.

### Drivers and role of retailers’ condoms selling behaviour

It is evident from this study that retailers’ selling behaviours have direct or indirect impact on accessibility and utilisation of condoms in the community. As illustrated (Table 4), at least 6 % of the retailers agreed with the customers who seemed to demand only one piece of condom mainly due to their ability to pay if not their limited knowledge that a single unit of condom could be risky to use several times without sterilization. Majority of scientific evidence warns that the improper use of condoms such as the tendency to share or reuse the same condom between several sexual actions done consecutively or between different sexual partners is risky. In fact, scientific guidelines recommend use of one condom once per one sexual action [42]. The testimony given by the retailers while confirming the experience shared by the barmaids and guesthouse workers interviewed in this study concerning the time of opening/closing the retail units sometimes causing unexpected inconvenience to potential condom customers is another practical demand drawback or barrier to actual utilization of condoms in the study communities. This together with the occasional shortage of condoms (especially the female condoms) evidently impedes accessibility and therefore utilization of condoms in the study communities, as found in other districts in Tanzania [29].

Moreover, it is sad that still there are potential users of condoms in the community who expressed doubt about condoms’ ability to protect users against HIV infections. The stigma attached to use of condoms is likely to be socially or culturally rooted and widespread in the community in Tanzania [8], as elsewhere in SSA [41, 43] and therefore not just an accidental viewpoint of the present study respondents. The reported experience by retailers that some customers (adults and adolescents alike) do not like to be known or seen while buying condoms at retail outlets, therefore, enforcing the sellers to abide by the requirements of such type of customers is another insight about the prevailing social stigma against condoms and extra-marital sexual relationships among the adults or premature sex among the adolescents [29]. These findings alert the AIDS control program authorities to prioritise repackaging and targeting right messages for different population groups within and between district settings.

### Strengths and limitations of the present study in relation to the existing knowledge

The present study provides useful information related to the supply and demand for condoms and operational challenges associated with condom promotion and use for HIV/AIDS prevention in Tanzania. Having involved a research team not directly responsible for planning and managing HIV/AIDS interventions, the study presents the findings that are not biased to the personal experiences or interest of the investigators. Also, the findings obtained especially with regard to the observed differences in experiences and behaviours of study population groups with different demographic characteristics are consistent with those obtained by previous researchers in other parts of Tanzania [28, 39, 44]. However, as any other study, the present one if not free of limitations or weaknesses [45], for instance, (1) the findings were obtained from two districts only out of over 120 districts in the country, meaning that there limitation relating to generalizability of the results presented for the whole country; (2) the results presented herein are based on self-reported sexual behaviour and this could be a deficiency in the study design since self-reported sexual behaviour may be inaccurate to such factors as poor recall, misunderstanding, and/or intentionally given false statements in attempt to avoid anticipated criticism (particularly if the investigators were not much trusted by the respondent) [29]; (3) the shying away of some of the respondents may be associated with the technique employed for data collection since face-to-face interviews exploring about sensitive issues relating to health and particularly the reproductive or sexual health may be inappropriate in some contexts especially where the social stigma against discussing such issues openly is high [46].

## Conclusion and policy implications

It is evident from this study that socio-economic factors related to knowledge and perceptions about condoms coupled with systemic constraints relating to limited supply of condoms have negative implications on the demand for and ultimate utilisation of condoms in Tanzania. Therefore, more concerted measures are necessary including those addressing the socio-economic and systemic challenges. The present study calls for possible, feasible, practicable and integrated approaches through multi-sector frameworks coupled with a sustained support to experts or teams opting for carrying out basic or operational research that seem to have potential and substantial contributions to the already exiting evidence that can lead to better informed policy decisions at global, regional, national, district and community levels. Meanwhile, the policy message informed by the condom gap revealed by the present study, as noted elsewhere in SSA [47] prompt us to see the need for program strategies that would help to increase the supply/distribution and promotion of both female and male condoms as well as promoting male circumcision especially in non-Islam dominated cultural societies meanwhile complementing or augmenting the already established biomedical interventions involving the use of ARV drugs and encouragement of voluntary counselling and testing for HIV in the society.

## Additional files

10.1186/s13104-015-1621-y A Structured Questionnaire for Data Collection from Barmaids and Guesthouse workers on the Study on Determinants of the Demand for Condoms in Mpwapwa and Mbeya Rural Districts, Tanzania.
10.1186/s13104-015-1621-y A Structured Questionnaire for Data Collection from Condom Retailers on Determinants of the Demand for Condoms in Mpwapwa and Mbeya Rural Districts, Tanzania.
