# Supplementary material for: Determinants of demand for condoms to prevent HIV infections among barmaids and guesthouse workers in two districts, Tanzania
Source: BMC Res Notes. 2015 Nov 2;8:630. doi: 10.1186/s13104-015-1621-y (PMC4630838; doi:10.1186/s13104-015-1621-y)
Supplement: Supplementary file 1 — 10.1186/s13104-015-1621-y A Structured Questionnaire for Data Collection from Barmaids and Guesthouse workers on the Study on Determinants of the Demand for Condoms in Mpwapwa and Mbeya Rural Districts, Tanzania. [file 13104_2015_1621_MOESM1_ESM.docx]

**Appendix 9.1.2 STRUCTURED INTERVIEW WITH BARMAIDS AND GUEST-HOUSE-WORKERS IN A TWO DISTRICT STUDY, TANZANIA**

**Investigator’s introduction:** Start with greetings. Then welcome the person you have approached for interview. Mention your name or designation. Tell what you are there for. Use the informed consent form as a reference and make sure it is only signed by those who have received your explanation on what study is about and have expressed their willingness to sign the informed consent form. Those who decline participation should also be thanked for their time to give you their ears and please don’t be disappointed for their decline.

**________________________________________________________________________**

**BACKGROUND INFORMATION**

(a) **Study Area**

District’s name……………………………………

Division’s name…….…………………………….

Ward’s name………………………………………

Village/Street’s name….………………………….

(b) **Respondent**

ID Number…………………………

1. Sex: **1**. Male **2**. Female
2. Age (in number of years): ……………………………..
3. Class (e.g. V, VI, VII)………………………
4. Parents’ information:
5. Education of father/male guardian……..…………………………………
6. Education of mother/female guardian…………………………………….
7. Occupation of father/male guardian……………………………………….
8. Occupation of mother/female guardian…………………………………….

**Sexual behavior, Awareness about condoms and attitudes towards condoms**

**(*For questions with pre-coded answers, please tick accordingly*)**

1. Do you know what sexual intercourse is about?

**1**. Yes  **2.** No

2. Have you ever participated in any sexual intercourse?

**1**. Yes **2.** No

3. At what age did you start participating in sexual intercourse?

**Age in years** _______________________________

4. What drove you participate in sexual intercourse?

(i) My own desire for sex **1**. Yes **2**. No

(ii) I was convinced/deceived by the partner **1**. Yes **2**. No

(iii) I was rapped by the partner/other people **1**. Yes **2**. No

1. Other causes (specify)………………………………………………………

…………………………………………………………………………………………

5. Have you ever heard about condoms?

**1**. Yes

**2**. No

6. How did you know about condoms?..................................................................................

7. What are condoms used for? (explain)………………………………………………..

8. Have you ever seen a condom yourself?

**1**. Yes

**2**. No

9. Have you ever used condom(s) yourself during sexual intercourse(s)?

**1**. Yes

**2**. No

**3**. Can’t say (my secret)

10. Do you have a sex partner?

**1**. Yes

**2**. No

**3**. Can’t say

11. Do you have one permanent sexual partner?

**1**. Yes

**2**. No

**3**. Can’t say

12. How frequently do you practice sexual intercourse with your partner?

**1**. Regularly (many times for a short time interval e.g. daily, weekly, monthly)

**2**. Occasionally (not regularly e.g. once in every 2+ months)

**3**. Can’t say (my secret)

13. Why haven’t you used condoms during sexual intercourse(s)?.............……………

14. If one needs condoms, where can they be found?......................................................

15. Are there condoms useful for men only or women only or both the men and women?

**1**. Men

**2**. Women

**3**. Both men and women

**4**. Not so sure (uncertain)

16. What names of the condoms do you currently know?............................................

……………………………………………………………………………………………

17. If you were asked to express your preference to condoms that are available at various sources, which one(s) would you identify?

Salama Condom **1**. Yes **2**. No

Dume **1**. Yes **2**. No

Other (specify)………………………………………………………………

18. To what extent do you agree with what is said about condom use being protective against the risks of infections through sexual intercourse?

**1**. Strongly agree

**2**. Agree

**3**. Disagree

**4**. Not certain

19. Do you think condoms should continue being promoted and distributed to children of your age – whether those in school or out of school?

**1**. Yes **2**. No **3.** Difficult to say

20. Why do you think (doubt) that condoms should not be promoted and distributed to be reached by the children of your age or a little younger age?.........………………………………………………………………………………

**Pricing, distribution, affordability, promotion and accessibility of condoms**

21. Are there condoms that can be accessed for free at various places where they are distributed?

**1**. Yes

**2**. No

**3**. Don’t Know (not sure/probably/Think so)

22. Are there condoms sold by various agencies to people needing them around this place?

**1**. Yes

**2**. No

**3**. Don’t Know

23. For condoms that are being sold, what is your view regarding their price at retail markets in terms of their affordability to people of your age?

1. Reasonable (affordable)
2. Expensive (higher than expected)
3. Other (specify)…………………………………………………………………………..

24. What experience do you have regarding condoms availability so that they can be accessed any time by people needing them around this place?

Widely available and all the time accessed **1**. Yes **2**. No

Sometimes the supply is lower than demand **1**. Yes **2**. No

Don’t have experience to say anything **1**. Yes **2**. No

Other views (specify)………………………………………………………..

25. To what extent are you pleased with the way the various agencies promote condoms e.g. advertising through mass media, street billboards, mobile vans, etc. effectively encourage people of your age see the importance of condom use?

**1**. Strongly pleased

**2**. Pleased

**3**. Not much pleased

**4**. Difficult to say

26. Do you think provision of AIDS education to children of your age including teaching them on such methods as use of condoms to prevent HIV/AIDS infections is desirable?

(i) To you as children? **1**. Yes **2**. No

(ii) To your parents/guardians to hear about? **1**. Yes **2**. No

**Retailers’ influence and Psychosocial Determinants of Condoms use**

27. What an you comment regarding the retailers’ behavior in selling condoms around this place in relation to the following dimensions:

(i) Language to customers coming to buy condoms

**1**. Acceptable

**2**. Sometimes disappointing

**3**. Don’t know

(ii) Way of stocking/storing the condoms e.g. in the shelves

**1**. Acceptable

**2**. Sometimes disappointing

**3**. Don’t know

(iii) Time of opening their shops/kiosks/outlets

**1**. Acceptable

**2**. Sometimes disappointing

**3**. Don’t know

(iv) Any other comment?.........................................................................................

28. How would you feel if you were noted by your parent/guardian holding or buying a condom?

1. No doubt at all (feel easy)
2. Shying away (shameful)
3. Fear (get worried)
4. Throw away or abandon buying the condom

29. Suppose you were noted by an adult member of your community other than your parent/guardian holding or buying a condom, would you feel the same way as you would feel if you were noted by your parents?

**1**. Yes

**2**. No

**3**. It depends

30. If you were noted by a child of your age or a little younger one who knows you buying or holding a condom, would you feel the same way you would feel if you were noted by an adult person?

**1**. Yes

**2**. No

**3**. It depends

THANKS VERY MUCH FOR YOUR TIME TO PARTICIPATE IN THIS STUDY AND ALL THE BEST IN YOUR INTENTIONS, PLANS AND ENDEAVORS
