# Supplementary material for: Determinants of demand for condoms to prevent HIV infections among barmaids and guesthouse workers in two districts, Tanzania
Source: BMC Res Notes. 2015 Nov 2;8:630. doi: 10.1186/s13104-015-1621-y (PMC4630838; doi:10.1186/s13104-015-1621-y)
Supplement: Supplementary file 2 — 10.1186/s13104-015-1621-y A Structured Questionnaire for Data Collection from Condom Retailers on Determinants of the Demand for Condoms in Mpwapwa and Mbeya Rural Districts, Tanzania. [file 13104_2015_1621_MOESM2_ESM.docx]

**Appendix 9.1.3 STRUCTURED QUESTIONNAIRE FOR THE RETAILERS**

**Investigator’s introduction:** Start with greetings. Then welcome the person you have approached for interview. Mention your name or designation. Tell what you are there for. Use the informed consent form as a reference and make sure it is only signed by those who have received your explanation on the study and their willingness to sign an informed consent form to confirm their voluntariness to participate in the study. Those who decline participation in the study should also be thanked for giving you their ears to listen what you wanted from them. Please, don’t be disappointed with their decline.

**________________________________________________________________________**

**BACKGROUND INFORMATION**

(a) **Study Area**

District’s name……………………………………

Division’s name…….…………………………….

Ward’s name………………………………………

Village/Street’s name….………………………….

(b) **Respondent**

Respondent’s ID:…………………………………………………………………………………..

Respondent’s type/category: **1**. Kiosk dealer

**2**. Shopkeeper

**3**. Medical Store (Cold drug shop)

Respondent’s age (in years):_______________________

Respondent’s sex: **1**. Male **2**. Female

Respondent’s highest education: **1**. Primary

**2**. Secondary

**3**. Higher than secondary (e.g. college)

**4**. Never gone to school

**Condom varieties, pricing, distribution, promotion and selling behavior**

1. Do you deal with condom business at your outlet? **1**. Yes **2**. No

2. Do you deal with male condoms alone or female condom alone or both?

**1**. Male alone

**2**. Female alone

**3**. Both

3. What specific varieties of condoms do you deal with at your outlet?

(i) Dume **1**. Yes **2**. No

(ii) Salama **1.** Yes **2**. No

(iii) Other (specify)…………………………………………………………………….

4. What motivated you decide to deal with condom selling business?.......................................

5. At what time do you mostly open your outlet for starting your daily business?.....................

6. At what time in do you close your outlet for business each day?...........................................

7. At what time during your daily business do you mostly experience customers coming to buy condoms at your outlet? **1**. early morning times

**2**. around mid-days

**3**. around afternoons

**4**. at evening times before darkness

**5**. after darkness has entered around night times

**6**. there is no specific time (It depends)

**7**. other (specify)……………………………………………

8. If you were to categorize in terms of age, who are your main customers in relation to the condoms you sell? **1**. Adolescents

**2**. Youths

**3**. Middle adults

**4**. Senior Adults

**5**. Have not cared to keep record/Can’t remember

9. If you were to categorize the sex, who are your main customers to the condoms you sell?

**1**. Males

**2**. Females

**3**. No clear picture (changing pattern of customer inflows)

**4**. Have not taken it seriously to take note/Can’t remember

10. What strategies do you use to promote your condom business to harness your potential customers or maintain your target customers of condoms?.................................................................................

11. Does your outlet happen to run out of stock of condoms to meet the existing demand?

**1**. Yes

**2**. No

12. What specifically causes your outlet to run out of supply of condoms?............................................

13. Is there a particular season or period of the year in which the demand for condom is higher than the rest of the seasons/periods? **1**. Yes **2**. No **3**. Have not seriously cared

14. In which particular season do you experience higher demand for the condoms you sell?................

15. How do you sell your condoms - on unitary basis or on packet (with several condoms) basis?

1. strictly on unitary basis
2. strictly on packet basis
3. either on unitary or on packet basis (depends on customers’ preference)

16. From you experience, do the customers you mostly meet for condom business prefer taking one condom or several condoms at once?

1. one condom at once
2. several condoms at once
3. have not taken it seriously to keep this in mind
4. other experience (specify)………………………………………………………

17. How do you compare the salability of the condoms and other health products e.g. drugs you may have at your outlet?

1. condoms are more saleable
2. other products are more saleable
3. no clear pattern separating between the two
4. have not taken it seriously to note the difference
5. other experience (specify)………………………………………………………

18. What price do you charge per a condom at your outlet?

1. Salama **Tshs**.________________
2. Dume: **Tshs**._________________
3. Other: **Tshs**.__________________

19. Have you ever experienced a customer begging you to offer a condom for free on ground of having no money to pay for it at your outlet in the last six months?

**1**. Yes **2**. No

20. How often have you been experiencing such kind of customers asking for free condoms in the last six months?

**1**. rarely **2**. frequently **3**. Only once

21. Which category would you put such customer(s) who begged you for free condoms?

1. adolescent male
2. youth male
3. adult male
4. adolescent female
5. youth female
6. adult female

**Psychosocial determinants of condom use or non-use behavior in the community**

22. If you were to rate, to what extent do the people coming to buy the condoms at your outlet do it openly with confidence even if they are seen by other people or secretly in fear of being seen?

1. all of them are confident and open
2. most of them are confident and open
3. The majority express shyness to be seen
4. All of them express shyness of being seen
5. Have not taken it seriously to note this

23. If you were to compare the different groups of population – adolescents, youths, middle adults and senior adults, which of these seem more being confident when coming for condoms at your place?

**1**. adolescents

**2**. youths

**3.** middle adults

**4**. senior adults

**5**. Have not taken it seriously to note this

THANK YOU VERY MUCH FOR YOUR COOPERATION AND ALL THE BEST IN YOUR BUSINESS.

**_________ ______________________________________________________________**
